# Breast cancer-specific mutations in CK1ε inhibit Wnt/β-catenin and activate the Wnt/Rac1/JNK and NFAT pathways to decrease cell adhesion and promote cell migration

**DOI:** 10.1186/bcr2581

**Published:** 2010-05-27

**Authors:** Silvie Foldynová-Trantírková, Petra Sekyrová, Kateřina Tmejová, Eva Brumovská, Ondřej Bernatík, Wulf Blankenfeldt, Pavel Krejčí, Alois Kozubík, Tomáš Doležal, Lukáš Trantírek, Vítězslav Bryja

**Affiliations:** 1 Biology Centre AS CR, v.v.i. AND University of South Bohemia, Branisovska 31, 37005 Ceske Budejovice, Czech Republic; 2 Institute of Experimental Biology, Faculty of Science, Masaryk University, Kotlarska 2, CZ-61137 Brno, Czech Republic; 3 Department of Cytokinetics, Institute of Biophysics AS CR, Kralovopolska 135, 60200 Brno, Czech Republic; 4 Max-Planck-Institute of Molecular Physiology, Otto-Hahn-Straße 11, 44227 Dortmund, Germany; 5 Current address: Department of Chemistry, Faculty of Science, Utrecht University, Padualaan 8, NL-3584 CH Utrecht, The Netherlands

## Abstract

**Introduction:**

Breast cancer is one of the most common types of cancer in women. One of the genes that were found mutated in breast cancer is casein kinase 1 epsilon (CK1ε). Because CK1ε is a crucial regulator of the Wnt signaling cascades, we determined how these CK1ε mutations interfere with the Wnt pathway and affect the behavior of epithelial breast cancer cell lines.

**Methods:**

We performed *in silico *modeling of various mutations and analyzed the kinase activity of the CK1ε mutants both *in vitro *and *in vivo*. Furthermore, we used reporter and small GTPase assays to identify how mutation of CK1ε affects different branches of the Wnt signaling pathway. Based on these results, we employed cell adhesion and cell migration assays in MCF7 cells to demonstrate a crucial role for CK1ε in these processes.

**Results:**

*In silico *modeling and *in vivo *data showed that autophosphorylation at Thr 44, a site adjacent to the breast cancer point mutations in the N-terminal lobe of human CK1ε, is involved in positive regulation of the CK1ε activity. Our data further demonstrate that, in mammalian cells, mutated forms of CK1ε failed to affect the intracellular localization and phosphorylation of Dvl2; we were able to demonstrate that CK1ε mutants were unable to enhance Dvl-induced TCF/LEF-mediated transcription, that CK1ε mutants acted as loss-of-function in the Wnt/β-catenin pathway, and that CK1ε mutants activated the noncanonical Wnt/Rac-1 and NFAT pathways, similar to pharmacological inhibitors of CK1. In line with these findings, inhibition of CK1 promoted cell migration as well as decreased cell adhesion and E-cadherin expression in the breast cancer-derived cell line MCF7.

**Conclusions:**

In summary, these data suggest that the mutations of CK1ε found in breast cancer can suppress Wnt/β-catenin as well as promote the Wnt/Rac-1/JNK and Wnt/NFAT pathways, thus contributing to breast cancer development via effects on cell adhesion and migration. In terms of molecular mechanism, our data indicate that the breast cancer point mutations in the N-terminal lobe of CK1ε, which are correlated with decreased phosphorylation activities of mutated forms of CK1ε both *in vitro *and *in vivo*, interfere with positive autophosphorylation at Thr 44.

## Introduction

Mammary carcinomas are one of the most common neoplasias in women. Several improvements in understanding the molecular pathology of breast cancer have been achieved in the past decade. In most cases, however, the molecular mechanisms underlying this malignancy are still unknown.

Sequencing of mammary carcinoma samples by Fuja and colleagues revealed that the casein kinase 1 epsilon (CK1ε) gene was mutated in this disease; CK1ε was found to be mutated within its N-terminal region with approximately 15% incidence [1]. CK1ε is a Ser/Thr kinase with many known functions and substrates. CK1ε phosphorylates several regulators of crucial processes, such as cell proliferation, differentiation, migration, and circadian rhythms. The key known targets of CK1ε involve p53, key components of the circadian clock, the Wnt signaling pathway, and cell division machinery (for a review, see [2]). In the original sequencing study, 19 nonsynonymous mutations were identified in the CK1ε gene in ductal carcinoma samples [1]. The identified mutations were shown to have a significant association with the loss of heterozygosity and decreased staining of CK1ε in the tumor sections. Some of the mutations in CK1ε were found repeatedly in several patients, such as L39Q (detected five times), L49Q (detected three times), and S101R (detected twice) [1]. These observations suggest that these mutations may affect CK1ε function and may be favored during the microevolution of the tumor, and thus may contribute to tumor progression.

Importantly, nothing is known about how these mutations affect the kinase activity and signaling potential of CK1ε and the behavior of mammary cells. In the present study, we characterized three CK1ε mutants that were previously identified in mammary carcinoma. We demonstrated that these CK1ε mutants had limited kinase activity and failed to phosphorylate the physiological targets of CK1ε *in vitro *and *in vivo*. The analyzed mutations acted as loss of function in the Wnt/β-catenin pathway and promoted the alternative Wnt/Rac1 pathway, which in turn decreased cell adhesion and promoted cell migration.

## Materials and methods

### Plasmids

ORFs of the wild-type (WT), full-length human CKIε cDNA (residues 1 to 416), two mutants mimicking either nonphosphorylatable Thr 44 (Thr44Ala) or constitutively phosphorylated Thr 44 (Thr44Asp), and three mutated versions (P3, P4, and P6) were cloned into pcDNA3. The truncated versions of CKIεΔC (residues 1 to 315) were cloned into pHAK-B3. Plasmids encoding mDvl2-Myc [3] and human Dvl3-Flag [4] have been previously described. Details and bacterial overexpression vectors are presented in Additional file 1.

### Structural modeling

The three-dimensional model for CK1ε was obtained via template-based homology modeling using the program PHYRE [5]. The mutated sites and kinase-specific functional domains were mapped onto a three-dimensional model of CK1ε using the program CHIMERA [6]. The kinase-specific functional domains in CK1ε were predicted using the NCBI Conserved Domain Database [7]. Predictions of changes in protein stability upon point mutations were conducted using CUPSTAT [8]. (Auto)Phosphorylation sites were predicted using GPS v. 2.1 [9].

### Western blot analysis, immunoprecipitation, and small GTPase activity assays

Western blot analysis, immunoprecipitation, and small GTPase activity assays were performed as previously described [10,11]. The antibodies used for the western blot analysis were as follows: mouse anti-Flag (M2; Sigma, Schnelldorf, Germany), goat anti-CK1e (sc6471; Santa Cruz Biotechnology, Heidelberg, Germany), mouse anti-Myc (sc-40) and anti-actin (sc-1615-R) (both Santa Cruz Biotechnology), anti-HA (HA.11; Nordic Biosite, Täby, Sweden), mouse anti-Rac (05-389; Upstate, Waltham, Massachusetts, USA), and mouse anti-Cdc42 (610928, 1:1,000; BD Biosciences, San Jose, California, USA). The antibodies used for immunoprecipitation were as follows: anti-CK1ε (sc-6471; Santa Cruz Biotechnology), anti-MYC (C3965; Sigma), and anti-FLAG (F1804; Sigma).

### Immunohistochemistry

Transfected cells were grown on glass coverslips, washed with PBS, fixed for 15 minutes in 4% paraformaldehyde, washed with PBS, and blocked in 1.5% BSA, 0.1% Triton X-100 in PBS for 1 hour. After overnight incubation at 4°C with the primary antibody, cells were washed with PBS, 0.1% Triton X-100, incubated for 2 hours at room temperature with secondary antibody, washed with PBS, 0.1% Triton X-100, and counterstained with 4 μg/ml 4'',6 - Diamidino - 2 - phenylindole, dihydrochloride. *Phalloidin*-Alexa488 (1:66; Molecular Probes, Carlsbad, CA, USA) was added in the last 30 minutes of incubation with the secondary antibody. Samples were analyzed with a FV1000 confocal microscope (Olympus). The following antibodies were used: mouse anti-Myc (1:150) and goat anti-CK1ε (1:100) (both from Santa Cruz Biotechnology), anti-goat-Cy5 (1:500; Molecular Probes), and anti-mouse-Alexa488 (1:1,000; Molecular Probes).

### Real-time impedance measurements

AceaE-plates^®^96 were used for non-invasive real-time measurements with an xCELLigence RTCA SP system and RTCA software version 1.1 (both Roche Applied Science, Indianapolis, IN, USA). First, a background measurement was performed using 100 μl complete cultivation media with or without CK1 inhibitors incubated for 30 minutes in the incubator (37°C, 5% CO_2_). MCF7 cells were trypsinized, quantified, and seeded (15,000 cells/well) in an additional 100 μl cultivation media. The impedance was then monitored continually for a period of 20 hours. Data are presented as a cell index. In parallel to xCELLigence measurements, cells were seeded (15,000 cells/well) in 24-well plates, and the effects of IC261 on cell number and cell viability were determined after 20 hours. The cell number was measured using a Coulter Counter (Immunotech a. s., A Beckman Coulter Company, Praque, Czech Republic). Cell viability was determined using eosin staining.

### Hanging drop assay

MCF7 cells (4 × 10^5 ^cells/ml) with or without CK1ε inhibitors were seeded in 30 μl drops on the inner side of a 10 cm plate lid. The lid of the plate was then turned upside down and placed on top of the plate filled with 10 ml PBS. MCF7 cells under the force of gravity aggregated at the bottom of the hanging drop. After 24 hours, cell clusters were photographed, collected, and resuspended by pipetting up and down seven times, and single cells released from the clusters were counted.

### Luciferase assays

HEK293 and MCF7 cells were transfected in a 24-well plate format with the indicated combinations of plasmids using polyethylene-imine (HEK293) or FuGENE (MCF7). The amounts used per well were as follows: vectors encoding Dvl2, Dvl3, and CK1ε, 300 ng; pRLtkLuc (Renilla Promega, Madison, WI, USA), 50 ng; and firefly luciferase reporters, 200 ng. The following luciferase reporters were used: pSuperTopFlash [12], p-E-cadherin-Luc [13], pNFAT-Luc, and pAP1-Luc (Stratagene, La Jolla, CA, USA). The total amount of plasmid DNA per well was kept constant in all conditions. Twenty-four hours post transfection, cells were harvested and processed using the Dual Luciferase kit (Promega, Madison, WI, USA) according to the manufacturer's protocol. To normalize for the efficiency of transfection, firefly luciferase values were normalized to Renilla luciferase in each well.

Each data point was run in duplicate, and at least three independent experiments were performed. Datasets from each experiment were normalized to the control, and all individually normalized experiments were statistically analyzed by analysis of variance and Tukey's multiple comparison post tests (GraphPad Prism software, La Jolla, CA, USA The graphs indicate the mean ± standard deviations from at least three independent replicates.

### Transwell assay

MCF7 cells (5 × 10^4 ^cells) were seeded into the upper chamber of Transwell plates (Transwell 96-well plate, 5.0 μm polycarbonate membrane; Corning (Corning, MY, USA)). The inhibitors (D4476 or IC261; Calbiochem, Darmstadt, Germany) were added into the bottom chamber at the indicated concentrations. Identical amounts of dimethylsulfoxide were used as a control. After 24 hours, cells that migrated through the filter into the bottom chamber were counted with a Coulter Counter. The number of migrating cells was normalized to control conditions and expressed as a migration index.

## Results

### CK1ε mutants can bind but fail to phosphorylate Dishevelled

**Figure 1:**
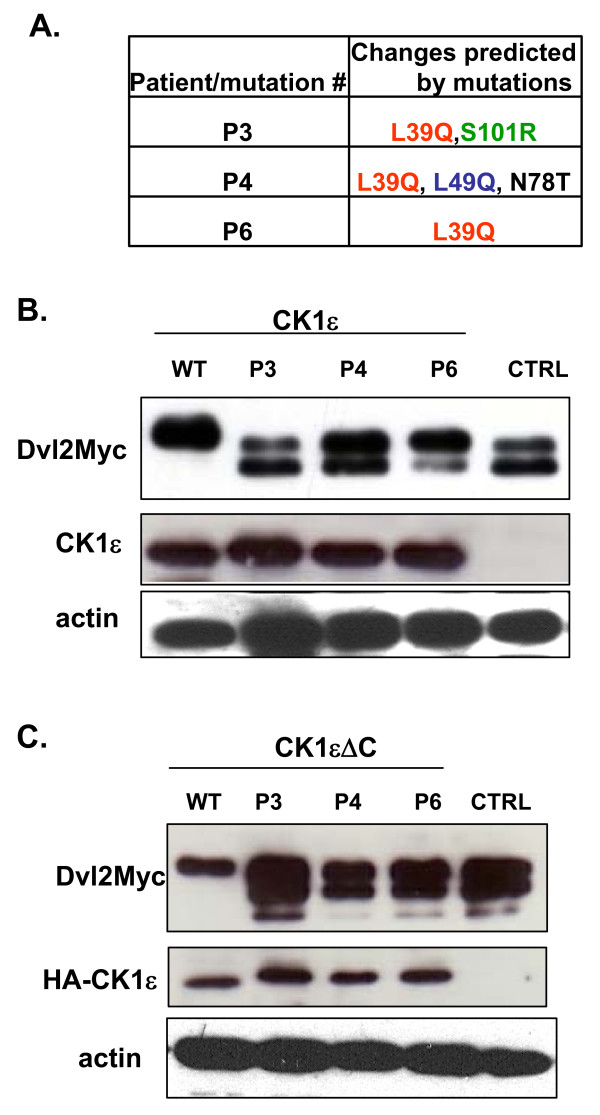
**Casein kinase 1 epsilon mutant phosphorylation of Dishevelled**. All three mutants of casein kinase 1 epsilon (CK1ε) bind Dvl but are unable to cause the typical phosphorylation-dependent shift of the Dvl protein. **(a) **Schematic representation of the CK1ε mutants used in this study. **(b) **HEK293 cells were transfected with a plasmid encoding Dvl2-Myc and the CK1ε variants. CK1ε phosphorylates Dvl proteins, which subsequently exhibit a mobility shift in western blot analysis. The P6 mutant maintains residual activity, while the P3 and P4 mutants are unable to promote a Dvl2-Myc shift. **(c) **Truncated versions of CK1ε that lack the C-terminal autoinhibitory domain do not differ from full-length proteins in their ability to shift the Dvl2 protein.

L39Q is the most frequently occurring mutation found in patients with breast cancer. In these patients, this mutation occurs alone (Patient P6) or in combination with other mutations - L39Q and S101R (Patient P3), or L39Q, L49Q, and N78T (Patient P4) (Figure 1a). Leu 39 and Leu 49, as well as Ser 101 (occupied exclusively by Ser or Thr), are absolutely conserved among CK1 paralogs and CK1ε orthologs (data not shown).

**Figure 2:**
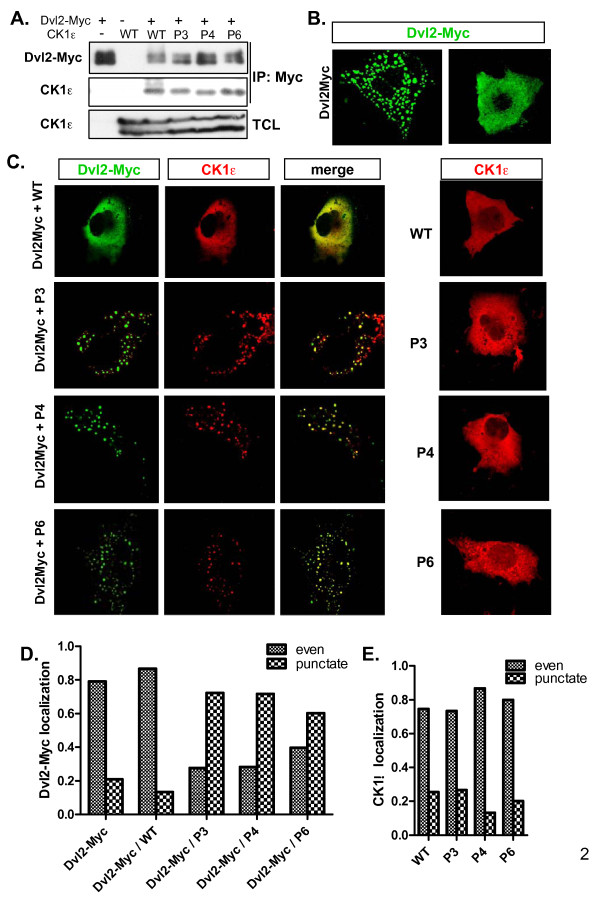
**Interaction of casein kinase 1 epsilon mutants and Dishevelled 2**. Casein kinase 1 epsilon (CK1ε) mutants associate with Dvl2, co-localize with Dvl2, and promote the punctate cytoplasmic localization of Dvl2. **(a) **HEK293 cell lysates transfected with either wild-type (WT) CK1ε or the P3, P4, and P6 mutants together with Dvl2-Myc were lysed and immunoprecipitated using an anti-Myc antibody. WT CK1ε and all of the CK1ε mutants efficiently bind the Dvl2 protein. **(b) **Dvl2-Myc protein localization in transfected COS7 cells was observed by confocal microscopy using an anti-Myc antibody. Dvl2 is either found in cytoplasmic inclusions or evenly dispersed within the cytoplasm. **(c) **WT CK1ε co-transfected with Dvl2-Myc into COS7 cells dissolves most of the Dvl2 punctae, resulting in the predominance of evenly distributed Dvl2 protein. In contrast, the P3, P4, and P6 mutants enhance the formation of Dvl2 clusters with a punctate appearance in the cytoplasm. A localization pattern of CK1ε was observed with an anti-CK1e antibody. All CK1ε forms show even cytoplasmic distribution when transfected alone. **(d), (e) **Cells with an even or punctate distribution of Dvl2 and CK1e were counted. For each condition, at least 150 cells were examined.

One of the best defined functions of CK1ε is the Wnt-induced phosphorylation of Dvl, a crucial component of the Wnt signaling cascades [11,14-20]. In the first step, we analyzed the capacity of individual CK1ε mutants to phosphorylate Dvl in mammalian HEK293 cells. As shown in Figure 1b, WT CK1ε promoted the formation of phosphorylated and shifted (PS) Dvl2, whereas mutant forms did not. A partial shift was observed with the P6 mutant, but the P3/P4 mutants were indistinguishable when compared with the control-transfected sample. Deletion of the C-terminus of CK1e, which inhibits CK1e kinase activity when phosphorylated [20], did not affect these results (Figure 1c). Importantly, using immunoprecipitation we were able to detect the presence of overexpressed Dvl2 or Dvl3, and both WT and mutated CK1e kinases in one protein complex (Figure 2a and Additional file 2), suggesting that the CK1e mutants still possess the ability to bind Dvl.

Phosphorylation of Dvl by CK1ε leads not only to the formation of PS-Dvl but also to changes in the intracellular distribution of Dvl [11,19]. Dvl is usually present in dynamic multiprotein aggregates [21,22] called Dvl dots. Based on the cellular context and activity of CK1e, which promotes the dissolution of Dvl aggregates, Dvl is usually present either in dots or in an even distribution (see Figure 2b for COS7 cells; see Additional file 2 for HEK293 cells). WT CK1ε strongly promoted an even localization of Dvl2-Myc (Figure 2c, d and Additional file 2). In contrast, all of the analyzed mutants (P3, P4, and P6) promoted the formation/maintenance of Dvl dots in COS7 cells and co-localized with Dvl in these multiprotein complexes (Figure 2c, d), similar to earlier observations with dominant negative CK1ε or CK1 inhibitors [11,19]. All CK1ε proteins were evenly distributed in the absence of Dvl (Figure 2c, e). These data together show that despite the fact that CK1ε mutants bind and co-localize with Dvl, they cannot phosphorylate Dvl or efficiently promote its even localization.

### *In silico *modeling of ductal carcinoma-specific CK1ε mutations

**Figure 3:**
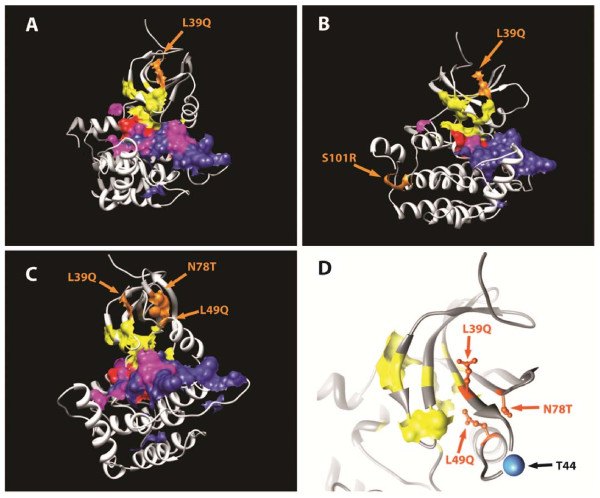
**Homology model of the catalytic domain of casein kinase 1 epsilon mutants**. Three-dimensional representation of a homology model of the catalytic domain of casein kinase 1 epsilon (CK1ε) (residues 1 to 295). Phylogenetically conserved functional sites across the serine/threonine kinase superfamily are indicated: pink, substrate binding pocket; yellow, ATP binding site; red, catalytic loop; blue, activation loop. The individual point mutations that were found in patients **(a) **P6 (L39Q), **(b) **P3 (L39Q, S101R), and **(c) **P4 (L39Q, L49Q, N78T) are indicated in orange. **(d) **A mutation cluster region in P4. The mutations and ATP binding region are indicated in orange and yellow, respectively. A blue sphere indicates a predicted autophosphorylation site (Thr 44).

To understand how individual mutations are spatially related to functionally conserved regions in CK1ε, we developed three-dimensional models for individual CK1ε mutants. In the single-point mutant (P6), the mutated site (Leu 39) directly adjoins conserved residues that participate in ATP binding (Figure 3a). The CK1ε mutant P3 contains two mutations, L39Q and S101R. These mutated sites are distant from each other not only in sequence but also spatially (Figure 3b). While Leu 39 is located in the β-strand-rich N-terminal lobe, Ser 101 is located at the C-terminal end of helix I in the primarily helical C-terminal subdomain. The S101R mutation is predicted to destabilize and alter local protein structure, thus affecting the structural integrity of the four-helix bundle, which forms the structurally conserved core of the C-terminal subdomain [23].

The last analyzed mutant, P4, contains three point mutations: L39Q, L49Q, and N78T. Although the individually mutated sites are distant in primary sequence, when mapped onto the three-dimensional structure of CK1ε they cluster into a very small area with a radius <10 Å that is located between strands four and five as well as into α-helix B of the N-terminal lobe (Figure 3c). Interestingly, all of these mutations surround a conserved predicted autophosphorylation site at Thr 44 in the N-terminal catalytic domain. This site is localized within a loop region between the terminus of the fourth β-strand (only five residues upstream from the mutation site L39Q) and α-helix B (five residues downstream from the second mutation site L49Q) (Figure 3d).

To assess kinase activities of the individual CK1ε mutants, we attempted to heterologously express individual mutants of human CK1ε as affinity tagged recombinant proteins lacking the autoinhibitory domain in *Escherichia coli*. Despite significant efforts, we were unable to obtain soluble overexpression for all of the constructs when we expressed them with the same affinity tag. Soluble expressions were feasible for WT, P3, P4, and P6, however, when the recombinant proteins were tagged to His_6_, to maltose binding protein, to maltose binding protein, and to Small Ubiquitin-like Modifier (SUMO), respectively. The individual recombinant fusion proteins were assayed for their ability to phosphorylate Dvl *in vitro*. Although the data from this *in vitro *phosphorylation assay were in qualitative agreement with our *in vivo *data (see above), we considered these *in vitro *data inconclusive since the presence of multiple different tags has made direct interpretation impossible (see Additional files 1 and 3).

### CK1ε mutants act as loss-of-function in the Wnt/β-catenin pathway

**Figure 4:**
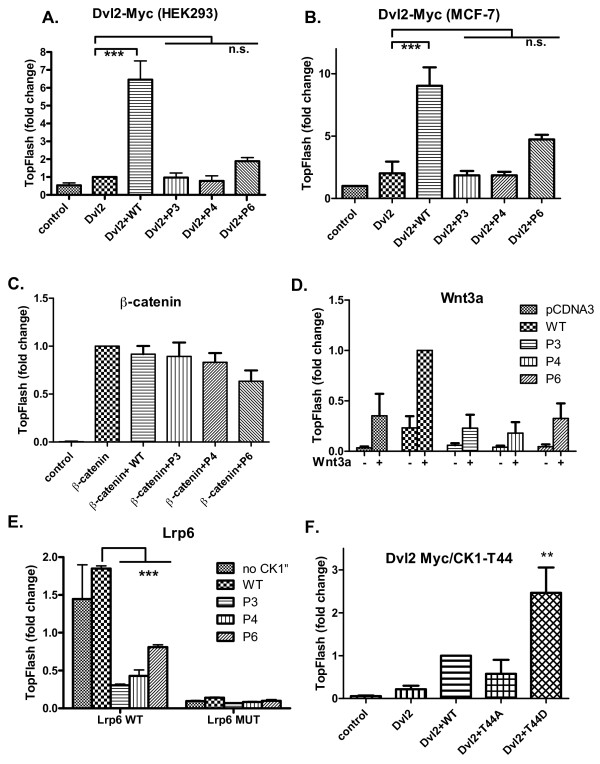
**Casein kinase 1 epsilon mutants downregulate Wnt/β-catenin signaling at the level of the Dishevelled protein**. HEK293 or MCF7 cells were transfected with the indicated plasmids, and after 24 hours TCF/LEF-driven transcription was measured using the SuperTopFlash dual luciferase reporter assay. **(a) **Dvl2 enhances TCF/LEF-dependent transcription when co-transfected with wild-type (WT) casein kinase 1 epsilon (CK1ε) in HEK293 cells. In contrast, all CK1ε mutants are unable to promote Dvl-mediated TopFlash. **(b) **Similar results to those obtained for HEK293 cells have been obtained for the breast cancer cell line MCF7. **(c) **β-Catenin induces the TCF/LEF transcriptional response in the cell by recruiting transcriptional cofactors directly to the TCF/LEF complex. Neither WT CK1ε nor the CK1ε mutants could influence the high TopFlash signal caused by constitutively active β-catenin, confirming that CK1ε and its mutants operate upstream of β-catenin (HEK293 cells). **(d) **The Wnt signaling pathway was induced by seeding B1A-wnt3a cells (fibroblasts that constitutively produce the Wnt3a protein) over transfected HEK293 cells. Wnt3a induces the Wnt/β-catenin/TCF/LEF pathway in control cells transfected with empty plasmid. The Wnt3-induced signal is enhanced by overexpression of WT CK1ε but is strongly depleted by overexpression of the CK1ε mutants. **(e) **Constitutively activation of the co-receptor LRP6 in transfected HEK293 cells confirmed the regulatory function of CK1ε downstream of the Wnt-receptor complex. WT CK1ε transduces the signal from the activated receptor towards TCF/LEF-driven transcription, while mutant CK1ε proteins abolish downstream signaling. Neither WT nor mutant CK1ε are able to markedly activate the TCF/LEF signal when Wnt stimulation is blocked by the mutant LRP6. **(f) **Nonphosphorylatable (T44A) and phospho-mimicking (T44D) mutants of CK1ε were co-expressed with Dvl2-Myc in HEK293 cells. The effects on TCF/LEF-dependent transcription show that T44 D behaves as overactive CK1ε. (a) to (f) Data represent the mean ± standard deviation from normalized values. *P < 0.05, ***P < 0.001; n.s., not significant; one-way analysis of variance, Tukey post-test, n ≥ 3.

To test the role of mutated CK1ε proteins in the canonical Wnt pathway, we induced Wnt/β-catenin signaling via the overexpression of several of the components of this pathway - such as Dvl2, Dvl3, β-catenin, Wnt3a, and the Lrp6 co-receptor - and analyzed TCF/LEF-driven transcription using the TopFlash reporter system [24]. As shown in Figure 4, Dvl2-Myc weakly activated the TopFlash reporter in HEK293 and MCF7 cells, but the signal was boosted when WT CK1e was co-expressed. The CK1e mutants P3 and P4 failed to synergize with Dvl2, and P6 promoted Dvl2-driven TopFlash only moderately (Figure 4a, b). Very similar results have been obtained with Dvl3-Flag (Additional file 4).

Importantly, the effects of CK1e were Dvl dependent because the overexpression of any form of CK1e had only negligible effects (Additional file 4). The inhibitory effects of the CK1e mutants were found at the level of Dvl because the activation of TopFlash by constitutively active (S33A)-β-catenin could not be significantly modulated by the overexpression of any CK1e (Figure 4c). Co-cultivation with fibroblasts producing Wnt3a or overexpression of crucial Wnt co-receptor Lrp6 efficiently induced TCF/LEF-dependent transcription. Co-expression of WT CK1e promoted TopFlash even further, whereas the expression of the P3, P4, and P6 CK1e mutants were able to reduce the Wnt3a/Lrp6-induced signal. This effect was obvious in Wnt-3a-stimulated cells and became statistically significant in cells overexpressing Lrp6 (Figure 4d, e). These analyses demonstrate that the P3, P4, and P6 mutants of CK1e are dysfunctional in the Wnt/β-catenin pathway and act upstream of β-catenin.

Our structural modeling (Figure 3) suggested that CK1e mutations found in breast cancer may affect the predicted autophosphorylation site at Thr 44. To test the function of Thr 44 we generated two mutants mimicking either nonphosphorylatable Thr 44 by replacing Thr 44 for alanine (T44A) or constitutively phosphorylated Thr 44 by replacing Thr 44 for aspartic acid (T44D). As shown in Figure 4f, T44D-CK1e is a more potent activator and T44A a less potent activator of TCF/LEF-driven transcription than WT CK1e when co-expressed with Dvl2-Myc. This finding confirms the positive functional role of Thr 44 phosphorylation in the modulation of CK1e activity, and explains how L39Q mutation contributes to the diminishing of CK1e activity in the Wnt/β-catenin pathway.

### CK1ε mutants activate small GTPase Rac-1 and AP1-driven transcription

**Figure 5:**
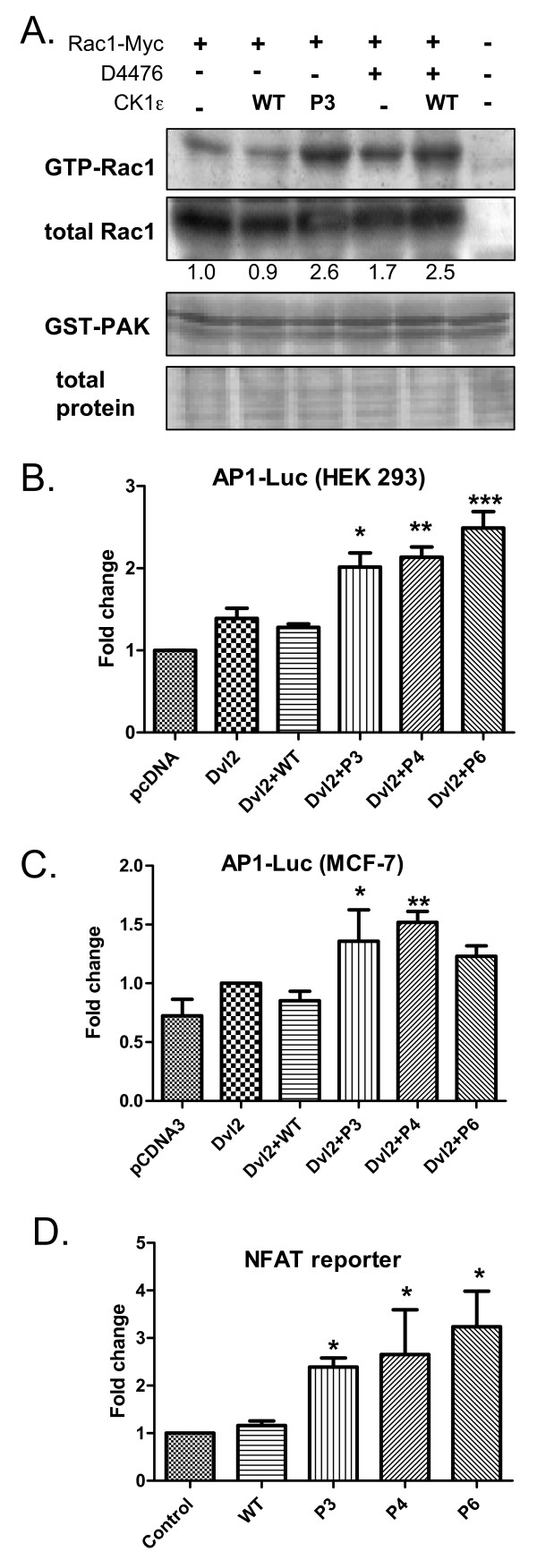
**Casein kinase 1 epsilon mutants act as loss of function in the Wnt/β-catenin pathway**. Casein kinase 1 epsilon (CK1ε) mutants activate small GTPase Rac1 and the transcriptional activity of AP1 and NFAT. **(a) **Lysates from HEK293 cells transfected with Rac1-Myc were subjected to pull-down of the active GTP-Rac1 form with agarose-PAK beads. HEK293 cells were either co-transfected with wild-type (WT) or mutant CK1ε or treated with 100 μM D4476 inhibitor for 4 hours prior to lysis. The P3 mutant promotes Rac1 activation, as a higher amount of GTP-Rac1 was pulled from the lysate (as compared with WT CK1ε). Consistently, D4476 inhibits the function of WT CK1ε and elevates the level of GTP-Rac1 in cells. Rac1 protein was detected with an anti-Myc antibody. Western blots were quantified by densitometry, and the GTP-Rac1/Rac1 ratios are indicated by the numbers below the panel. After probing, membranes were stained with amidoblack to confirm equal protein loading. **(b) **HEK293 cells or **(c) **MCF7 cells were transfected with an AP1-luciferase reporter, Dvl2-Myc, and CK1ε as indicated. Cells were lysed, and luciferase activity was analyzed the next day. **(d) **HEK293 cells were transfected with a NFAT-luciferase reporter and CK1ε as indicated. Cells were lysed, and luciferase activity was analyzed the next day. (b) to (d) Data represent the mean ± standard deviation. *P < 0.05, **P < 0.01; one-way analysis of variance, Tukey post-test, n ≥ 3.

Our laboratory and others have previously shown that CK1e can act as a switch that promotes Wnt signaling pathways that are dependent on PS-Dvl (such as the Wnt/β-catenin or PS-Dvl-dependent noncanonical pathways) and blocks the Wnt/Rac1/JNK pathway [10,19]. We hypothesized that the CK1e mutants may mimic CK1e inhibition. To test this prediction, we tested the effects of WT and P3 CK1e on Rac1 activation. As shown in Figure 5a, WT CK1e slightly downregulated Rac1 activity, whereas expression of the P3 mutant or inhibition of CK1 by D4476 promoted this activity. We were not able to detect any effects of CK1e on the activity of another small GTPase, Cdc42 (Additional file 5), and we also failed to detect active RhoA in HEK293 cells (data not shown).

Importantly, the CK1e mutants also promoted Dvl2-Myc-induced AP1-mediated transcription, which is downstream of the Rac1/JNK pathway (Figure 5b, c). Similar results were obtained with Dvl3-Flag (Additional file 4). We also tested the effects of the CK1e mutants on the noncanonical Wnt/Ca^2+ ^pathway. As shown in Figure 5d, WT CK1e did not affect the transcriptional activity of NFAT, a transcription factor that is activated by calcium waves. The activity of the NFAT reporter was promoted by the CK1e mutants, however, suggesting that mutant forms of CK1e can promote the Wnt/Ca^2+ ^pathway.

In summary, our data demonstrate that mutant CK1e, which is present in ductal carcinoma, can act as an inhibitor of the Wnt/β-catenin and an activator of the noncanonical Wnt/Rac1/JNK and Wnt/Ca^2+ ^pathways.

### CK1ε inhibition decreases cell adhesion and promotes migration in MCF7 cells

To date, whether mutations in CK1e that are associated with breast cancer have any effect on the behavior of breast adenocarcinoma cells is not clear. Our results show that mutated forms of CK1e behaved identically to CK1 inhibition in all tested assays - compare Figure 2 and [11], and compare Figure 5 and [10]. Based on these analyses, one could expect that the presence of CK1e mutants is functionally equivalent to the pharmacological inhibition of CK1e.

**Figure 6:**
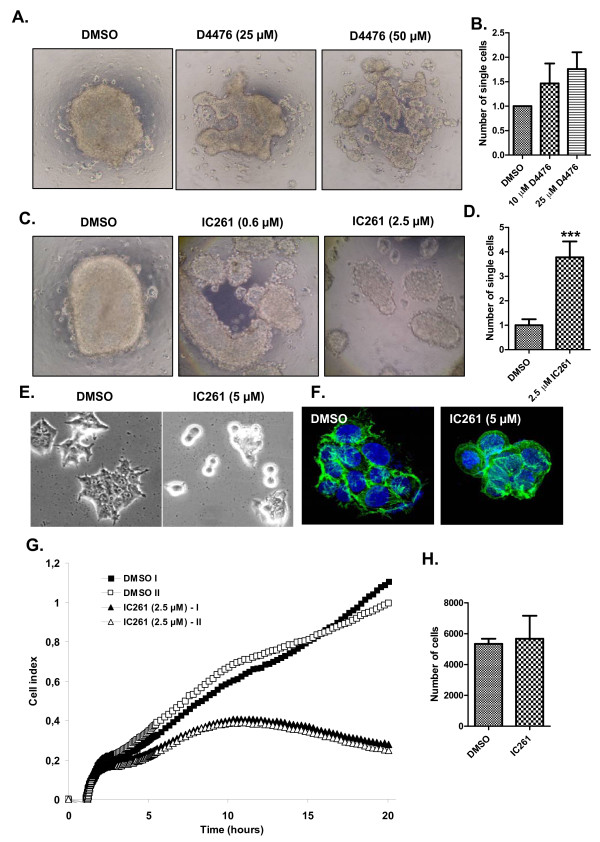
**Casein kinase 1 epsilon inhibition decreases the cell adhesion of MCF7 breast epithelial cells**. **(a) to (d) **MCF7 cells grown in hanging drop culture tend to form compact cell clusters under control conditions (dimethylsulfoxide (DMSO)). With increasing amounts of the casein kinase 1 epsilon (CK1ε) inhibitors - (a), (b) D4476, and (c), (d) IC261 - the large single-cluster formation is disrupted, and cells form smaller and disintegrated aggregates. After resuspending these aggregates by pipetting up and down seven times, a significantly higher number of single cells was observed to be released from clusters in conditions treated with (b) D4476 and (d) IC261, as normalized to control. ***P < 0.001, Student's *t *test, n = 3. **(e), (f) **MCF7 cells were seeded in the presence or absence of 5 μM IC261. Cell morphology was analyzed after 20 hours in (e) phase contrast or (f)after staining of actin filaments (phalloidin, green) and cell nuclei (Hoechst, blue). **(g), (h) **Dynamics of MCF7 cell adhesion was monitored for 20 hours using the xCELLigence System (Roche Applied Science). Cell adhesion is expressed as the cell index for two control wells (DMSO) and two experimental wells (2.5 μM IC261). (h) Treatment with IC261 did not affect the cell number (compared with control) during 20 hours of stimulation.

Taking advantage of this finding, we tested the effects of the CK1 inhibitors D4476 and IC261 on the cell adhesion of MCF7 epithelial breast cancer cells. The MCF7 cell line retains several characteristics of differentiated mammary epithelium, including the ability to process estradiol via cytoplasmic estrogen receptors and the capability of forming domes [25], and is suitable for analyzing cellular changes during tumor progression. D4476 is the most specific inhibitor of CK1 known to date [26,27], whereas IC261 specifically inhibits the CK1δ and CK1ε isoforms at doses <10 μM [27,28]. MCF7 cells efficiently aggregated in hanging drops and usually formed one cell cluster per drop (Figure 6a) after overnight aggregation. Inhibition of CK1 interfered with aggregate formation and caused the MCF7 cells to become more loosely attached to each other (Figure 6a, c). To quantify the level of adhesion, we mechanically resuspended the cell clusters and counted the number of single cells, which was increased by CK1 inhibition (Figure 6b, d). Similar results were obtained when we downregulated the levels of CK1δ and CK1ε in MCF7 cells via siRNA-mediated knockdown (Additional file 6). Next, we seeded the MCF7 cells in the presence of 5 μM IC261 and compared the morphology of the adherent cells after 20 hours. Control cells efficiently adhered to the plastic surface, spread out, and formed cytoskeletal actin networks that were visible as stress fibers and cytoplasmic protrusions (Figure 6e, f). In contrast, IC261-treated cells attached only loosely to the surface and exhibited a predominantly rounded morphology with no detectable stress fibers and protrusions (Figure 6e, f).

To quantify the intensity of cell adhesion to the cell surface, we employed direct measurements of impedance caused by cell adhesion using the xCELLigence System (Roche Applied Science). This technology measures changes in the electrical conductivity produced by cells, which attach to golden electrodes that cover the surface of the well. The changes correspond to the area of cell that is in direct contact with the bottom of the well. These parameters allow for quantification of adhesion intensity (termed cell index) given the prerequisite that cell number and cell viability are identical between samples. CK1 inhibition dramatically decreased the cell index (Figure 6g). Because 2.5 μM IC261 did not affect the cell number (Figure 6h) or cell viability (89% vs. 87.6% for dimethylsulfoxide vs. 2.5 μM IC261), we interpreted changes in the cell index as decreased adhesion to the dish surface. In summary, these results suggest inhibition of CK1δ/ε prevents the formation of cell-cell and cell-surface contacts in epithelial MCF7 cells.

**Figure 7:**
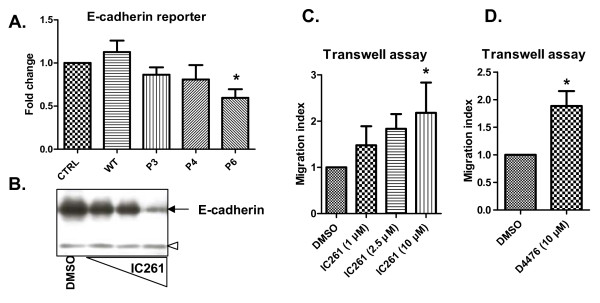
**Blocking casein kinase 1 epsilon function decreases E-cadherin expression and promotes cell migration**. **(a) **Wild-type (WT) and mutant casein kinase 1 epsilon (CK1ε) were expressed in MCF7 cells together with the reporter encoding E-cadherin-promoter coupled to luciferase. Cells were lysed and the activity of luciferase was analyzed next day. **(b) **MCF7 cells were treated after seeding out by increasing concentrations of IC261 (2.5 μM, 5 μM and 10 μM) for 48 hours and the expression of E-cadherin was analyzed by western blotting. Empty arrowhead, unspecific band, which confirms equal protein loading. **(c), (d) **MCF7 cells were seeded out in the upper part of the Transwell migration chamber and were stimulated with indicated doses of (c) IC261 and (d) D4476. The number of migrating cells in the bottom chamber was counted next day, and is expressed as the migration index. Data represent mean ± standard deviation. *P < 0.05; one-way analysis of variance; (a), (c)Tukey post test, (d) Student's *t *test; n = 3. DMSO, dimethylsulfoxide.

The decrease in cell adhesion that was induced by CK1 inhibition and observed in different experimental setups (Figure 6) might be relevant to cell behavior associated with tumor progression, such as epithelial-mesenchymal transition or increased cell migration. This view is supported by our analysis of the E-cadherin promoter (Figure 7a). E-cadherin mediates intracellular contacts and is commonly used as a marker of epithelial fate, whose downregulation contributes to a shift towards mesenchymal fate [29]. Overexpression of WT CK1e in MCF7 cells slightly increased the activity of the E-cadherin promoter. In contrast, mutant forms of CK1e decreased reporter activity, and the effects of the P6 mutant were statistically significant from WT (Figure 7a). Similar results were obtained in HEK293 cells (Additional file 7). These data suggest that mutation of CK1e contributes to decreased adhesion through the regulation of E-cadherin expression.

Treatment with the CK1e inhibitor IC261 decreased the expression of endogenous E-cadherin in a dose-dependent manner in MCF7 cells (Figure 7b). These changes in cell adhesion translate into changes of the migratory capacity of MCF7 cells in Transwell assays. As shown in Figure 7c, the dose-dependent inhibition of CK1δ/ε by IC261 promoted the migration of MCF7 cells. Similar effects were obtained with 10 μM D4476, which also increased the migration of MCF7 cells (Figure 7d). These results demonstrate that inhibition of CK1e or analogically mutants of CK1e can decrease cell adhesion and promote cell migration via mechanisms involving the activation of Wnt/Rac1 and Wnt/Ca^2+ ^and the repression of E-cadherin expression.

## Discussion

CK1ε is a crucial regulator of both the canonical Wnt/β-catenin pathway [14,15,17] and noncanonical Wnt pathways [10,11,30-32]. Wnt ligands from both classes - which either activate β-catenin or do not - activate CK1e, which in turn phosphorylates Dvl [11,20]. Phosphorylated PS-Dvl is thought to mediate downstream signaling, which results in the stabilization and nuclear translocation of β-catenin or in the activation of some noncanonical pathways [11,32].

Our results demonstrate that the three CK1ε mutants analyzed in this study that were identified in samples of breast cancer efficiently bind, but fail to phosphorylate, Dvl and act as loss of function in the Wnt/β-catenin pathway. We demonstrate that cells expressing these mutants are biased towards Rac1/JNK and NFAT activation at the expense of the Wnt/β-catenin pathway. Importantly, phosphorylation of Dvl by CK1e was shown to be inhibitory for the noncanonical Wnt/Dvl/Rac1/JNK pathway [10,19]. CK1e can thus act as a switch that directs signaling away from the Wnt/Rac1/JNK branch of noncanonical signaling towards the Wnt/β-catenin and PS-Dvl-dependent noncanonical pathways. Wnt/Ca^2+^, the other branch of noncanonical Wnt signaling, was shown to be antagonized by CK1 [33]. Our results confirm this observation and show that the CK1e mutants tested here increase the transcriptional activity of NFAT. These effects are not promoted by co-expression of Dvl (data not shown), suggesting that CK1e can repress NFAT directly. Indeed, phosphorylation by CK1e was shown to promote the cytoplasmic localization of NFAT and reduce its transcriptional activity in other experimental systems [34]. Together, our results provide the first evidence that the switch function of CK1ε, which was predicted based on *Xenopus *and cell culture experiments, is physiologically relevant and may contribute to cancer progression in ductal carcinoma.

The observation that ductal carcinoma-specific mutants of CK1ε promote the Wnt/Rac1/JNK and NFAT pathways and, on the other hand, inhibit the Wnt/β-catenin pathway are in good agreement with some clinical observations. The expression of β-catenin in breast cancer is usually low and was shown by several laboratories to correlate with poor prognosis [35-38]. Moreover, β-catenin is not localized within the cell nucleus in breast tumors [35-38], suggesting that Wnt/β-catenin signaling is in the *off *state in most breast cancers. Important to note, however, is the fact that there are also reports supporting a positive role for Wnt/β-catenin signaling in breast cancer (for a review, see [39]).

Wnt itself was first identified as an oncogene that is activated by the insertion of the mouse mammary tumor virus; and the mouse mammary tumor virus-Wnt-1 transgenic mouse is a well-established model for studies of the genetic basis of breast cancer (for a review, see [40]). Moreover, increased nuclear β-catenin levels, which correlate with cyclin D_1 _levels and a poor prognosis, were found in a subset of patients [41]. In contrast to other cancers, such as colon or skin cancer, the key components of the Wnt/β-catenin pathway, such as axin, adenomatous polyposis coli, or β-catenin are mutated in only a small portion of cases (for a review, see [39]). The levels of Wnt pathway components, which are common to both the canonical and noncanonical pathways, are altered more often; Dvl1 is upregulated in 50% of ductal breast cancer cases [42], and sFRP1 - a soluble Wnt antagonist that can block both Wnt/β-catenin and noncanonical pathways - is repressed in more than 80% of breast carcinomas [43]. These observations suggest that modulators of Wnt signaling, both at the extracellular level (sFRP1) and intracellular level (Dvl and CK1ε), will have a critical role in the biological outcome.

CK1ε has been shown to be highly expressed in non-invading ductal carcinoma *in situ *[1], which is also highly positive for β-catenin staining [36]. Based on our findings, we can speculate that the high activity of CK1ε in ductal carcinoma *in situ *reduces Rac-1/JNK and NFAT activity, keeping the cells tightly attached and blocking tumor invasion. CK1ε function can be compromised by somatic mutations [1], which dramatically affect CK1ε kinase activity (present study). The functional importance of this event supports the fact that samples with mutations in CK1ε show an increased frequency of loss of heterozygosity at the CSNK1ε locus [1]. Lack of CK1ε activity/expression leads to the activation of the Rac1/JNK/AP1 and NFAT pathways, which mediates the invasion of breast cancer cells and correlates with increased aggressiveness of the breast cancer [33,44-47]. Based on our data and other published information, we propose that mutation of CK1ε might be important for the transition between ductal carcinoma *in situ *and invasive carcinoma.

The effect of mutation or lack of CK1ε on Wnt signaling remains to be tested. In this context, it is especially important to determine how the status of CK1ε affects Wnt5a. Although Wnt5a is implicated in breast cancer pathology, its functional mechanism remains unclear. First, Wnt5a expression was shown to positively correlate with disease-free survival [48], and Wnt5a blocks breast cancer cell invasion [49-51]. On the other hand, Wnt5a is frequently upregulated in breast tumors in comparison with surrounding tissues [52] and was shown to promote the invasion of MCF7 cells via the JNK pathway [47]. We can speculate in cells with mutated CK1ε that Wnt5a will predominantly signal via the Rac-1/JNK and NFAT pathways, thus promoting breast cancer cell invasion and tumor aggressivity [44,45]. In contrast, in cells with abundant and intact CK1ε, Wnt5a principally stimulates other noncanonical signaling pathways that involve CK1, such as the Wnt/CK1ε/Rap1 [32] and Wnt/Yes-Cdc42-CK1α [33] pathways, and promotes cell adhesion [33].

In summary, our data demonstrate that the CK1ε mutants found in breast cancer act as loss of function, suppress jWnt/β-catenin, and promote Wnt/Rac-1-mediated and NFAT-mediated pathways. Furthermore, we show that inhibition of CK1ε reduces the intracellular adhesion and increases the migration of breast cancer cells. Our findings show that CK1ε has the potential to act as a tumor suppressor in breast cancer via its negative effects on the Wnt/Rac1/JNK and NFAT pathways. These results demonstrate for the first time how mutations in CK1ε affect cell behavior and may provide a general paradigm for consequences of CK1ε alteration in cancer.

## Conclusions

In the present study, we functionally analyzed mutations in CK1ε that are frequently found in breast cancer. Our data demonstrate that breast cancer-specific mutants of CK1ε act as loss of function in the Wnt/β-catenin pathway but activate the Wnt/Rac1/JNK and Wnt/Ca^2+ ^pathways. Physiological consequences of these signaling events in MCF7 breast cells include increased migratory capacity and decreased E-cadherin expression and cell adhesion.

## Abbreviations

BSA: bovine serum albumin; CKIε: casein kinase 1 epsilon; Dvl: Dishevelled; GST: glutathione *S*-transferase; ORF: open reading frame; PBS: phosphate-buffered saline; PS-Dvl: phosphorylated and shifted Dishevelled; siRNA: small interfering RNA; WT: wild type.

## Competing interests

The authors declare that they have no competing interests.

## Authors' contributions

SF-T, PS, KT, OB, and VB carried out the molecular genetic and biochemical studies. SF-T, EB, WB, and LT participated in cloning, plasmid production and protein purification. PK, AK, and TD designed and coordinated the study. LT and VB designed and coordinated the study, and drafted the manuscript. All authors read and approved the final manuscript.

## Supplementary Material

Additional file 1 **Supplementary materials and methods**. Materials and methods for construction of the recombinant protein expression constructs, for recombinant protein overexpression and purification, for *in vitro *kinase assays, for construction of vectors for mammalian expression, and for siRNA-mediated knockdown of CK1ε. Notes: pOPIN vectors [54] were used for bacterial overexpression. The GST-bPDZ construct was prepared as described previously [55]. siRNA-mediated knockdown performed as described previously [11]. Click here for file

Additional file 2 **Interaction between casein kinase 1 epsilon mutants and Dishevelled**. (a) HEK293 cell lysates transfected with either WT CK1ε or the P3, P4, and P6 mutants together with Dvl3-Flag were lysed and immunoprecipitated using an anti-Flag antibody. WT CK1ε and all of the CK1ε mutants efficiently bind the Dvl3 protein. (b) HEK293 cells were transfected with WT CK1ε or P3, P4 and P6 mutants together with Dvl2-Myc. Dvl2 protein localization in transfected HEK293 cells was observed by confocal microscopy using anti-Myc antibody. Dvl2 is either found in cytoplasmic inclusions or evenly dispersed within cytoplasm. Wt CK1ε co-transfected with Dvl2-Myc dissolves most of Dvl2 punctae, resulting in predominance of evenly distributed Dvl2 protein. In contrast, P3, P4 and P6 mutants are not able to promote even localization to the extent of WT CK1ε. The graphs indicate localization patterns (%) in 150 cells. Click here for file

Additional file 3 **Recombinant CK1εΔC mutants exhibit different kinase activity**. (a) His_6_-WT CK1εΔC phosphorylates the α and β isoforms of its natural substrate casein (1 to 3), while the kinase activity of the mutants maltose binding protein (MBP)-P3ΔC (L39Q, S101R) and MBP-P4ΔC (L39Q, L49Q, N78T) is strongly reduced (4 to 9). A single mutation in SUMO-P6ΔC (L39Q) results in partial kinase activity of the CK1ε enzyme (10 to 12). (b) The bPDZ domain of Dvl is phosphorylated by individual recombinant kinases similarly as casein. (c) The CK1ε target sequence in Dvl2, which corresponds to residues 145 to 168 of hDvl1 (ENLEPETETESVVSLRRERPRRR), was prepared as a synthetic peptide. This sequence was phosphorylated by His_6_-WT CK1εΔC and partially phosphorylated by the SUMO-P6ΔC mutant. The MBP-P3ΔC and MBP-P4ΔC mutants were unable to phosphorylate this peptide. Click here for file

Additional file 4 **Reporter assays with Dishevelled 3 protein**. TopFlash and AP1 luciferase assays with Dvl3 protein confirm results obtained with Dvl2. Graphs indicate the mean ± standard deviation from three independent replicates. (a) Co-expression of Dvl3-Flag and WT CK1ε in HEK293 potentiates Wnt/β-catenin signaling, while CK1ε mutants have opposite effects and downregulate TCF/LEF mediated luciferase transcription. (b) Co-expression of Dvl3-Flag and WT CK1ε in MCF7 potentiates Wnt/β-catenin signaling, while CK1ε mutants are unable to do so, similarly to the situation in HEK293 cells. (c) CK1ε forms without Dvl overexpression do not elevate TCF/LEF-dependent transcription as compared with control empty plasmid. (d) Dvl3-Flag and WT CK1ε transfected in HEK293 cells decrease JNK/AP1 signaling. In contrast, each mutant CK1ε together with Dvl3-Flag induces transcription from AP1 luciferase reporter. Click here for file

Additional file 5 **Casein kinase 1 epsilon does not activate Cdc42**. HEK293 cells were either transfected with CK1e forms or treated with 100 μM D4476 inhibitor 4 hours prior to lysis. Lysates from HEK293 cells were subjected to pull-down of active GTP-Cdc42 form with agarose-GST-WASP beads, which specifically interact only with the activated form of Cdc42. Amount of Cdc42 in pull-down (GTP-Cdc42) and in the original lysate (Cdc42) were detected by Cdc42 specific antibody using western blotting. Click here for file

Additional file 6 **siRNA-mediated knockdown of casein kinases decreases cell adhesion**. MCF7 cells were transfected with either control siRNA or mixture of siRNAs targeted against CK1δ and CK1ε, and were subjected to the hanging drop assay next day. Cells were photographed 24 hours after seeding; cell clusters with typical morphology are presented. Knockdown of CK1δ and CK1ε decreases cell adhesion, which leads to the formation of looser cell aggregates. The efficiency of knockdown of CK1ε has been determined by western blotting, actin used as a loading control. Click here for file

Additional file 7 **The effects of casein kinase 1 epsilon mutants on E-cadherin expression in HEK293 cells**. WT and mutant CK1ε were expressed in HEK cells together with the reporter encoding E-cadherin-promoter coupled to luciferase. Cells were lysed and the activity of firefly luciferase, which reflects the activity of E-cadherin promoter, was analyzed next day. Renilla luciferase was used as an internal control. All results were normalized to Renilla and to the control transfection. Graph shows mean ± standard error of the mean from three independent experiments. Click here for file
