# Supplementary material for: Breast cancer-specific mutations in CK1ε inhibit Wnt/β-catenin and activate the Wnt/Rac1/JNK and NFAT pathways to decrease cell adhesion and promote cell migration
Source: Breast Cancer Res. 2010 May 27;12(3):R30. doi: 10.1186/bcr2581 (PMC2917022; doi:10.1186/bcr2581)
Supplement: Additional file 1 — Supplementary materials and methods. Materials and methods for construction of the recombinant protein expression constructs, for recombinant protein overexpression and purification, for in vitro kinase assays, for construction of vectors for mammalian expression, and for siRNA-mediated knockdown of CK1ε. Notes: pOPIN vectors [54] were used for bacterial overexpression. The GST-bPDZ construct was prepared as described previously [55]. siRNA-mediated knockdown performed as described previously [11]. [file bcr2581-S1.PDF]

Construction of the expression constructs, protein over-expression and purification

The CKIε DNAs corresponding to truncated forms of CKIε lacking auto-inhibition domain in a forms of wild type and P3, P4, and P6 mutants were generated from full-lengths constructs, courtesy of Dr. C. Modak, by PCR. The constructs of CK1ε catalytical domain corresponding to wild-type, P3, P4, and P6 mutants were introduced via homologous recombination into four different bacterial expression vectors, pOPINE, pOPINF, pOPINM, and pOPINS producing C-terminally His_6-, N-terminally His_6-, N-terminally maltose binging protein-, and N-terminally SUMO-tagged fusion proteins, respectively. All PCR products were sequenced in their entirety. The vectors were introduced into the (DE3)RIL *E.coli* over-expression strain. Subsequently, the transfected bacteria were screened for soluble expression of the individual proteins under three different growth and induction conditions. Soluble over-expression of CKIεΔC wild-type was observed only for C-terminally His_6-tagged protein. Soluble P3ΔC and P4ΔC mutants were obtained in a form of N-terminally MBP-tagged fusion proteins. P6ΔC mutants provided soluble over-expression as N-terminally SUMO-tagged fusion protein.

The CKIe gene constructs were expressed in *E. coli* BL21(DE3) Codon Plus RIL cells (Novagen). An overnight culture from a single colony was diluted (1:100) into fresh Terrific Broth (TB) auto-induction medium supplemented with 50 mg l^{-1} ampicillin and 34 mg l^{-1} chloramphenicol. The cells were grown at 310 K in the TB medium with vigorous shaking for 4 hours. Then, the temperature was set to 298 K and auto-induced gene expression was allowed for 24 hours. The cells were harvested by centrifugation for 15 min at 6000*g*, resuspended in 50 m*M* Na_2HPO_4 pH 7.5 300 m*M* NaCl supplemented with 2 m*M* β-mercaptoethanol and then lysed with a fluidizer. The lysate was then centrifuged at 150 000*g* (Beckmann Optima L--70K ultracentrifuge; Ti-45 rotor) for 30 min and the resulting supernatant filtered, adsorbed and eluted from Ni–NTA affinity resin. Fractions containing CKIε fusion proteins, as judged by sodium dodecyl sulfate (SDS) gel electrophoresis, were pooled, dialyzed against 50 m*M* sodium phosphate buffer (pH=7) and 150 m*M* NaCl and concentrated prior to purification using gel filtration on Superdex 75 (GE Healthcare) in the same buffer. The purified protein was then transferred into the storage buffer (50 m*M* sodum phosphate buffer pH=8.0, 300 mM NaCl, 0.1% Tween, 10% glycerol) and stored at -80 ºC. For all mutants, any attempt to cleave off the tags resulted in the protein precipitation. Therefore, fusion, recombinant proteins (His_6-CKIεΔC, MBP-P3ΔC, MBP-P4ΔC, SUMO-P6ΔC) were purified and used in in vitro phosphorylation assays in the uncleaved form.

In vitro kinase assays

Kinase assays were performed with purified, human, truncated forms of CKIε that lacked their auto-inhibition domain (residues: 1-315), i.e., the recombinant fusion proteins: His_6-CKIεΔC, MBP-P3ΔC, MBP-P4ΔC, and SUMO-P6ΔC. The following CKIε substrates were used in the kinase assays: i) α+β and β caseins (Sigma: Cat. No. C5980 and C6950), ii) a purified GST-tagged bPDZ fusion protein, and iii) a synthetic peptide fragment (ENLEPETETESVVSLRRERPRRR) that corresponds to a specific domain of human Dvl 2 (residues: 145-168) (JPT Peptide Technologies). Reactions with a total volume 20 μl containing 5 μM kinase, 0.5 μl $^{[32]}$P ATP (MP Biomedicals, Cat. No. 38101X; 10 mCi/ml) in kinase buffer (75 μM ATP, 0.5 mM DTT, 7 mM $MgCl_2$, 100 μg/ml BSA, 30 mM HEPES, pH 7.5), and ~2 μg protein substrate were incubated for 30 minutes at 37ºC followed by SDS-PAGE analysis. Gels were stained with Coomassie solution, dried, and subjected to autoradiography.

Construction of vectors for mammalian expression

Open reading frames of human CKIε cDNA wt full-length (residues: 1-416) and three mutated versions (P3, P4, and P6) in pET vectors, were amplified by PCR using following specific primers: CKIεFW sense 5'-aagcttatggagctacgtgtggg-3', and CKIεRV antisense 5'-tctagattacttcccgagatggtcaa-3'. The PCR products were purified and inserted into pGEM-Teasy vector. All fragments were subsequently cloned into pcDNA3 vector by HindIII – XbaI sites. The truncated versions of CKIεΔC wt and P3ΔC, P4ΔC, and P6ΔC mutants (residues: 1-315) were amplified by PCR using primers CKIε-NFW sense 5'-aagcttgagctacgtgtgggg-3', and CKIε-NRV antisense 5'-tctagattacctctcctcgcgttcg-3', cloned into pGEM-T easy followed by subcloning at the HindIII/PstI site of pHAK-B3.

siRNA-mediated knockdown

Following siRNAs have been used: control (sc37007), CK1δ (sc29910) and CK1ε (sc29914) (all from Santa Cruz Biotechnology)
