# Supplementary figures and images for: Breast cancer-specific mutations in CK1ε inhibit Wnt/β-catenin and activate the Wnt/Rac1/JNK and NFAT pathways to decrease cell adhesion and promote cell migration
Source: Breast Cancer Res. 2010 May 27;12(3):R30. doi: 10.1186/bcr2581 (PMC2917022; doi:10.1186/bcr2581)

**A**

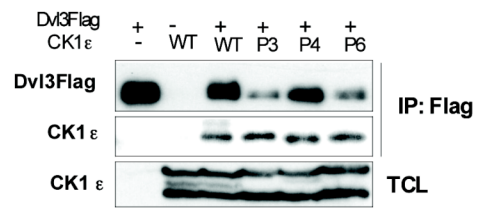

**B**

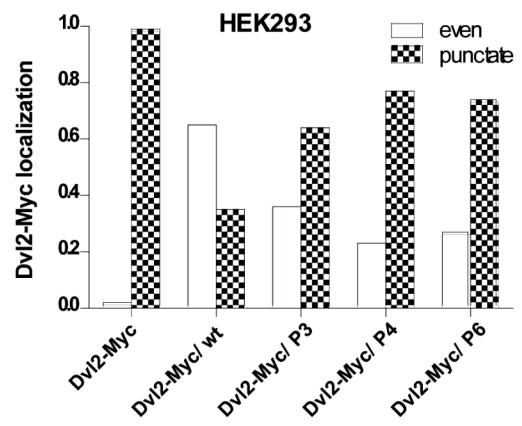

Supplement: Additional file 2 — Interaction between casein kinase 1 epsilon mutants and Dishevelled. (a) HEK293 cell lysates transfected with either WT CK1ε or the P3, P4, and P6 mutants together with Dvl3-Flag were lysed and immunoprecipitated using an anti-Flag antibody. WT CK1ε and all of the CK1ε mutants efficiently bind the Dvl3 protein. (b) HEK293 cells were transfected with WT CK1ε or P3, P4 and P6 mutants together with Dvl2-Myc. Dvl2 protein localization in transfected HEK293 cells was observed by confocal microscopy using anti-Myc antibody. Dvl2 is either found in cytoplasmic inclusions or evenly dispersed within cytoplasm. Wt CK1ε co-transfected with Dvl2-Myc dissolves most of Dvl2 punctae, resulting in predominance of evenly distributed Dvl2 protein. In contrast, P3, P4 and P6 mutants are not able to promote even localization to the extent of WT CK1ε. The graphs indicate localization patterns (%) in 150 cells. [file bcr2581-S2.PDF]

**A.**

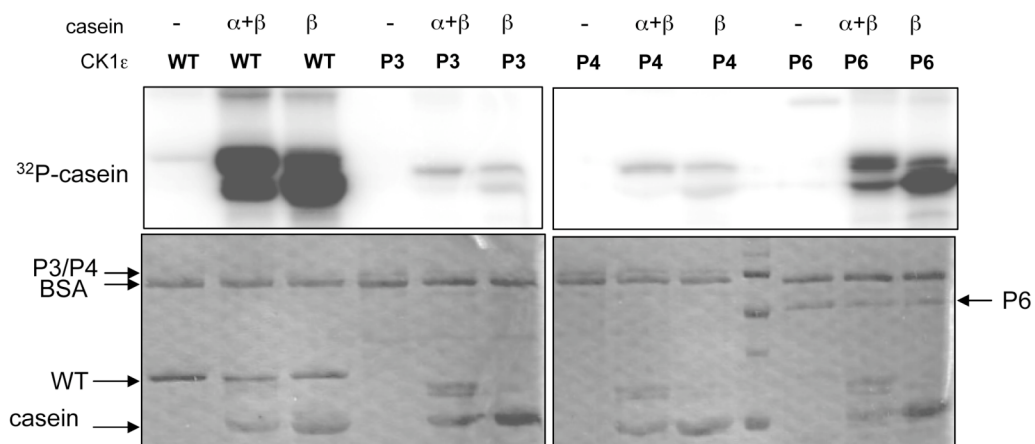

**B.**

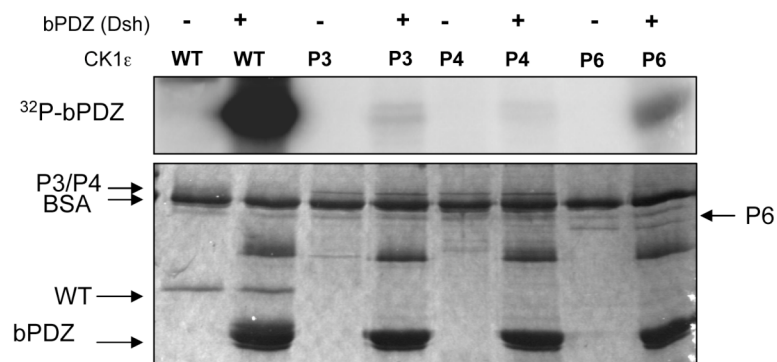

**C.**

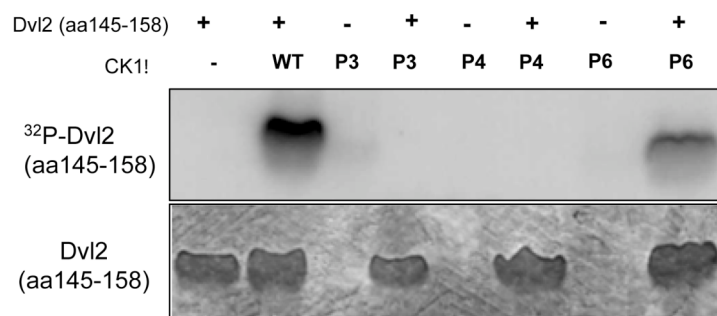

Supplement: Additional file 3 — Recombinant CK1εΔC mutants exhibit different kinase activity. (a) His6-WT CK1εΔC phosphorylates the α and β isoforms of its natural substrate casein (1 to 3), while the kinase activity of the mutants maltose binding protein (MBP)-P3ΔC (L39Q, S101R) and MBP-P4ΔC (L39Q, L49Q, N78T) is strongly reduced (4 to 9). A single mutation in SUMO-P6ΔC (L39Q) results in partial kinase activity of the CK1ε enzyme (10 to 12). (b) The bPDZ domain of Dvl is phosphorylated by individual recombinant kinases similarly as casein. (c) The CK1ε target sequence in Dvl2, which corresponds to residues 145 to 168 of hDvl1 (ENLEPETETESVVSLRRERPRRR), was prepared as a synthetic peptide. This sequence was phosphorylated by His6-WT CK1εΔC and partially phosphorylated by the SUMO-P6ΔC mutant. The MBP-P3ΔC and MBP-P4ΔC mutants were unable to phosphorylate this peptide. [file bcr2581-S3.PDF]

**A**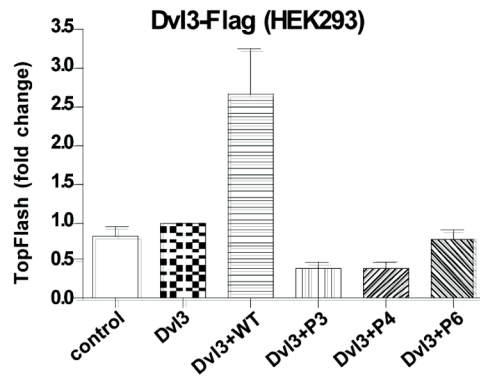**B**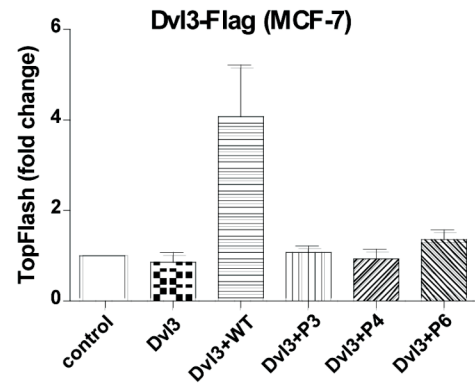**C**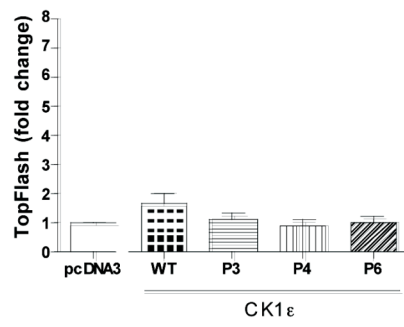**D**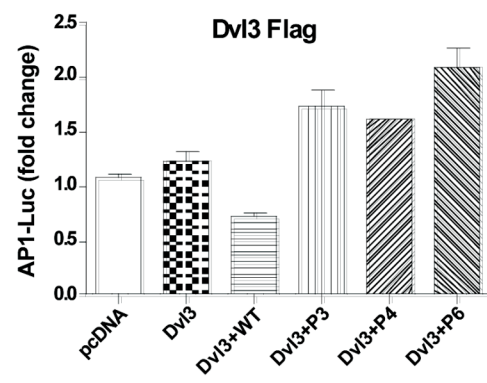

Supplement: Additional file 4 — Reporter assays with Dishevelled 3 protein. TopFlash and AP1 luciferase assays with Dvl3 protein confirm results obtained with Dvl2. Graphs indicate the mean ± standard deviation from three independent replicates. (a) Co-expression of Dvl3-Flag and WT CK1ε in HEK293 potentiates Wnt/β-catenin signaling, while CK1ε mutants have opposite effects and downregulate TCF/LEF mediated luciferase transcription. (b) Co-expression of Dvl3-Flag and WT CK1ε in MCF7 potentiates Wnt/β-catenin signaling, while CK1ε mutants are unable to do so, similarly to the situation in HEK293 cells. (c) CK1ε forms without Dvl overexpression do not elevate TCF/LEF-dependent transcription as compared with control empty plasmid. (d) Dvl3-Flag and WT CK1ε transfected in HEK293 cells decrease JNK/AP1 signaling. In contrast, each mutant CK1ε together with Dvl3-Flag induces transcription from AP1 luciferase reporter. [file bcr2581-S4.PDF]

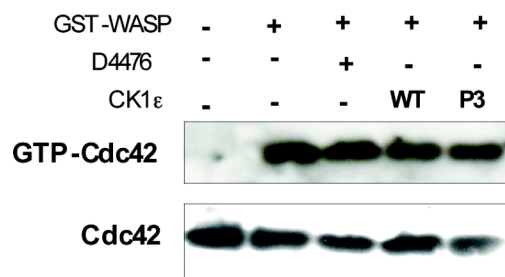

Supplement: Additional file 5 — Casein kinase 1 epsilon does not activate Cdc42. HEK293 cells were either transfected with CK1e forms or treated with 100 μM D4476 inhibitor 4 hours prior to lysis. Lysates from HEK293 cells were subjected to pull-down of active GTP-Cdc42 form with agarose-GST-WASP beads, which specifically interact only with the activated form of Cdc42. Amount of Cdc42 in pull-down (GTP-Cdc42) and in the original lysate (Cdc42) were detected by Cdc42 specific antibody using western blotting. [file bcr2581-S5.PDF]

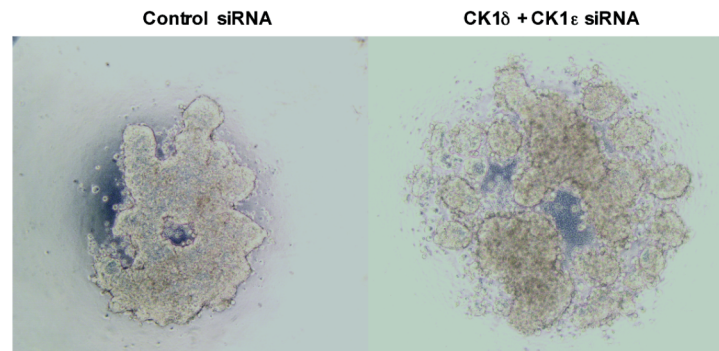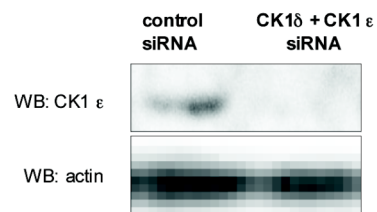

Supplement: Additional file 6 — siRNA-mediated knockdown of casein kinases decreases cell adhesion. MCF7 cells were transfected with either control siRNA or mixture of siRNAs targeted against CK1δ and CK1ε, and were subjected to the hanging drop assay next day. Cells were photographed 24 hours after seeding; cell clusters with typical morphology are presented. Knockdown of CK1δ and CK1ε decreases cell adhesion, which leads to the formation of looser cell aggregates. The efficiency of knockdown of CK1ε has been determined by western blotting, actin used as a loading control. [file bcr2581-S6.PDF]

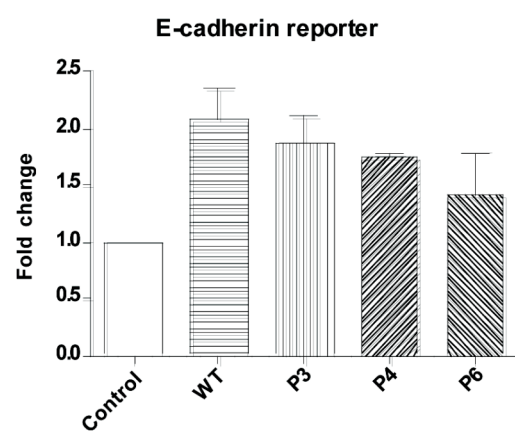

Supplement: Additional file 7 — The effects of casein kinase 1 epsilon mutants on E-cadherin expression in HEK293 cells. WT and mutant CK1ε were expressed in HEK cells together with the reporter encoding E-cadherin-promoter coupled to luciferase. Cells were lysed and the activity of firefly luciferase, which reflects the activity of E-cadherin promoter, was analyzed next day. Renilla luciferase was used as an internal control. All results were normalized to Renilla and to the control transfection. Graph shows mean ± standard error of the mean from three independent experiments. [file bcr2581-S7.PDF]
